# Design, synthesis and biological evaluation of immunostimulating mannosylated desmuramyl peptides

**DOI:** 10.3762/bjoc.15.174

**Published:** 2019-07-29

**Authors:** Rosana Ribić, Ranko Stojković, Lidija Milković, Mariastefania Antica, Marko Cigler, Srđanka Tomić

**Affiliations:** 1 University Center Varaždin, University North, Jurja Križanića 31b, HR-42000 Varaždin, Croatia; 2 Ruđer Bošković Institute, Bijenička cesta 54, HR-10000 Zagreb, Croatia; 3 Department of Chemistry, Technical University Munich, Lichtenbergstraße 4, D-85748 Garching, Germany; 4 Faculty of Science, University of Zagreb, Horvatovac 102a, HR-10000 Zagreb, Croatia

**Keywords:** adamantane, adjuvant activity, desmuramyl peptide, mannose

## Abstract

Muramyl dipeptide is the minimal structure of peptidoglycan with adjuvant properties. Replacement of the *N*-acetylmuramyl moiety and increase of lipophilicity are important approaches in the preparation of muramyl dipeptide analogues with improved pharmacological properties. Mannose receptors present on immunocompetent cells are pattern-recognition receptors and by mannose ligands binding they affect the immune system. Here we present the design, synthesis and biological evaluation of novel mannosylated desmuramyl peptide derivatives. Mannose was coupled to dipeptides containing a lipophilic adamantane on N- or C-terminus through a glycolyl or hydroxyisobutyryl linker. Adjuvant activities of synthesized compounds were investigated in the mouse model using ovalbumin as an antigen. Their activities were compared to the previously described mannosylated adamantane-containing desmuramyl peptide and peptidoglycan monomer. Tested compounds exhibited adjuvant activity and the strongest enhancement of IgG production was stimulated by compound **21** (Man-OCH_2_-ᴅ-(1-Ad)Gly-ʟ-Ala-ᴅ-isoGln).

## Introduction

**Figure 1:**
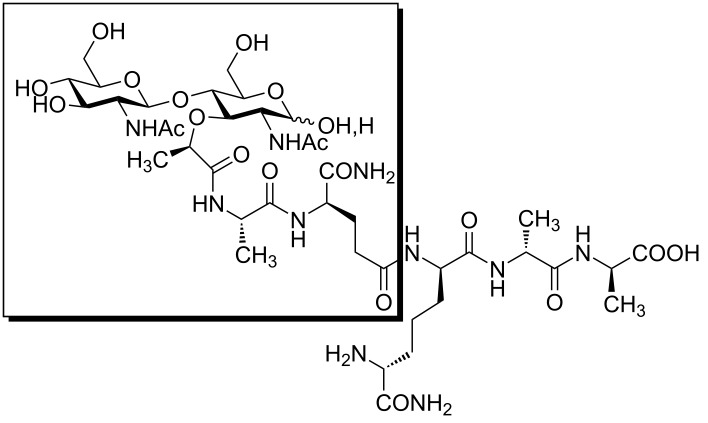
Peptidoglycan fragments with immunostimulating properties.

Dendritic cells capture and internalize invading pathogens. Pathogen-associated molecular patterns have been known for a long time to affect the immune system of mammalian hosts and therefore have been extensively studied as possible adjuvants for vaccines. Peptidoglycan is a polymeric component of the Gram-positive and Gram-negative bacterial cell wall. Breakdown products of polymeric peptidoglycan are called muropeptides. Muropeptides act as agonists of pathogen recognition receptors (PRRs) and therefore stimulate immune response and induce T cell differentiation [1–3]. They activate innate immune responses and contribute to the development of adaptive immunity. Immune response is initiated by the activation of PRRs located on the immune cell surface, by cytosolic or endosomal PRRs. PRRs are classified into: Toll-like receptors (TLRs), RIG-I-like receptors (RLRs), NOD-like receptors (NLRs) and C-type lectin receptors (CLRs) [4]. Muramyl dipeptide (MDP, *N*-acetylmuramyl-ʟ-alanyl-ᴅ-isoglutamine) is the smallest peptidoglycan fragment (Figure 1) capable of replacing the whole *Mycobacterium* in complete Freund’s adjuvant. MDP triggers an immune response by activating the mammalian NOD-like receptor, nucleotide binding oligomerization domain-containing protein 2 (NOD2). NOD2 is an intracellular protein that signals via the NF-κB pathway to proximally activate innate immunity through macrophage response as well as to more distally affect adaptive immunity through the production of antigen-specific T cells [5]. MDP binding to NOD2 has been confirmed [6] as well as the crystal structure of NOD2 in the inactive ADP-bound state [7]. MDP is the structural fragment of the peptidoglycan monomer (PGM, Figure 1) which is used in this work. PGM is a well-defined and characterized disaccharide pentapeptide, β-ᴅ-GlcNAc-(1→4)-ᴅ-MurNAc-ʟ-Ala-ᴅ-isoGln-*meso*DAP(εNH_2_)-ᴅ-Ala-ᴅ-Ala, originating from *Brevibacterium divaricatum* [8–9].

**Figure 2:**
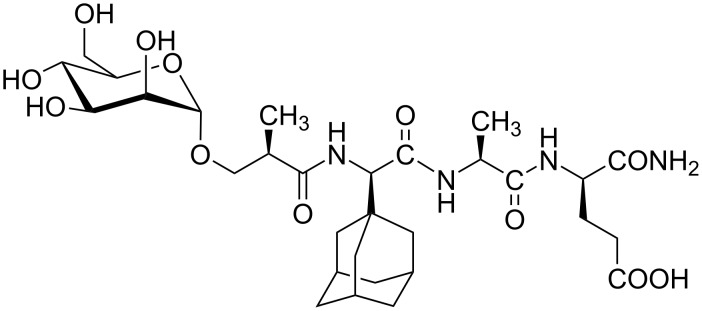
Immunostimulating mannosylated desmuramyl peptide (ManAdTP).

Peptidoglycans activate macrophages via TLR2 receptor, whereas MDP lacks TLR2-agonistic activity [10]. PGM and MDP have similar immunostimulating activity and they are reduced in comparison to the potent complete Freund’s adjuvant which is used as golden standard for adjuvant activity [11]. However, strong toxicity of complete Freund’s adjuvant disables its clinical application. MDP is too pyrogenic for clinical application as well and suﬀers from rapid elimination. Therefore, numerous MDP analogues and derivatives were synthesized, in order to improve the properties of the parent molecule [12–16]. Replacement of the *N*-acetylmuramyl moiety with various acyl groups represents an important approach in the design of new immunologically active MDP analogues [17]. MDP analogues lacking the *N*-acetylmuramyl group are called desmuramyl peptides. Structure–activity studies of the MDP derivatives and analogues suggest that the ʟ-Ala-ᴅ-isoGln pharmacophore is essential for the immunostimulatory properties but the introduction of lipophilic substituent into MDP analogues can increase its adjuvant activity [12,18]. Up to now, our research was directed towards isomeric desmuramyl peptides containing lipophilic unnatural amino acid, adamantylglycine (AdGly), bound to the N-terminus of ʟ-Ala-ᴅ-isoGln dipeptide part as well as their mannosylated derivatives. Different isomers of mannosylated adamantyl tripeptides, regarding the chiral centers introduced at adamantylglycine and spacer which connect the sugar part to the adamantyltripeptide were synthesized and biologically evaluated [19–21]. The best adjuvant activity in experiments in vivo showed the ManAdTP derivative (Figure 2) which has a ᴅ-configuration at the (adamant-1-yl)glycine moiety and (*R*)-configuration at the hydroxyisobutyryl linker. Its activity was higher than PGM that was used as reference compound.

The results indicate that introduction of mannose plays a significant role in stimulation of the immune response and the possibility of effecting the immune response by the mannose receptor family, group I CLRs, present on immunocompetent cells (such as macrophages and dendritic cells) [22–23]. It has been shown that uptake of liposomes displaying mannose ligands attached to the surface enhanced the uptake in human monocyte-derived dendritic cells [24].

**Figure 3:**
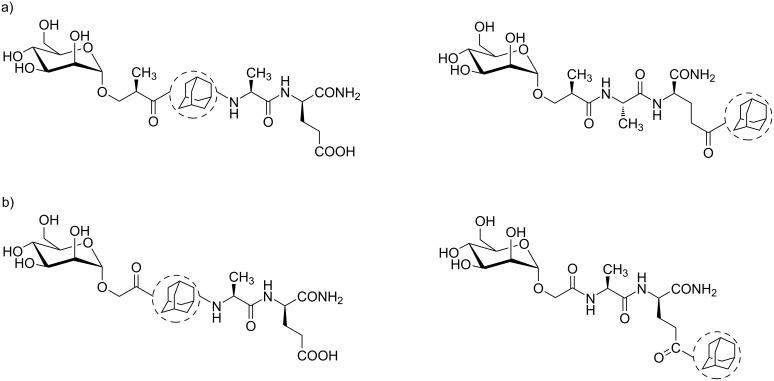
General structure of: a) glycolyl and b) (*R*)-hydroxyisobutyryl derivatives.

Here we describe a structure–activity relationship (SAR) study on novel mannosylated desmuramyl peptide derivatives in which two series of compounds were prepared: (i) derivatives containing a glycolyl linker between mannose and dipeptide, and (ii) derivatives containing a parent (*R*)-hydroxyisobutyryl linker (Figure 3). In both series, positions of adamantane binding (to the N- or C-terminus) were altered in comparison with derivatives lacking the adamantane moiety. Immunostimulating properties of synthesized derivatives were assessed in vivo using ovalbumin as an antigen.

## Results and Discussion

### Design

Desmuramyl peptides enter into the cell by passive absorption and this process depends on lipophilicity [25]. Numerous analogues and derivatives which incorporated different lipophilic groups have shown improved activity [14,18,21,26]. To enable the systematic investigation of influence of lipophilic adamantane on the immunostimulating activity, derivatives with altered position of the adamantane moiety (at the N- or C-terminus of the dipeptide), as well as derivatives lacking the adamantane moiety, were prepared. Furthermore, herein we describe a strategy for the preparation of two series of mannose MDP analogues based on the ManAdTP hit compound. The attachment of mannose to the desmuramyl dipeptide may contribute to the recognition of the adjuvant compound by specific mannose receptors expressed at immune cells. Mannosylated drug delivery systems enhance uptake and activation of dendritic cells and increase T cell proliferation [24,27–28]. Mannosylation of the adamantylated desmuramyl peptides amplified the adjuvant activity in experiments in vivo [20–21]. Two series of manno-conjugates were prepared: (i) derivatives with glycolyl linker and (ii) the ones with (*R*)-hydroxyisobutyryl linker which is present in the parent ManAdTP. The glycolic linker was introduced due to the fact that *N*-glycolyl muramyl peptides induce significantly higher activation of NOD2 than MDP [29–30]. Mycobacteria present in complete Freund’s adjuvant, and related Actinomycetes, produce *N*-glycolyl MDP by the hydroxylase action on MDP (*N*-acetylmuramic acid within the peptidoglycan). The influence of structural modifications on immunomodulating properties was estimated by the immunostimulatory effect on secondary humoral response to ovalbumin (antigen) in BALB/c mice.

### Chemistry

**Scheme 1:**
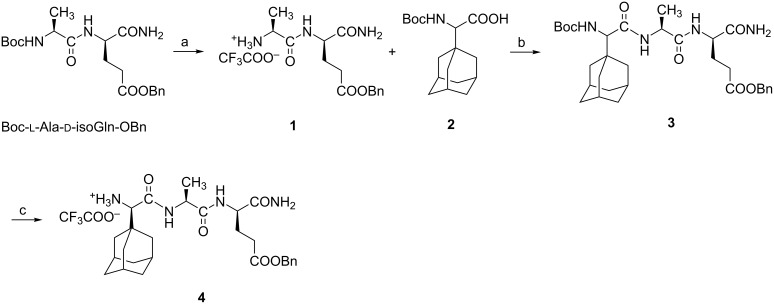
Synthesis of desmuramyl peptides modified at N-terminus. Reagents and conditions: a) TFA/DCM 1:2, rt, 1 h, quantitative; b) EDC·HCl, HOBt·H_2_O, Et_3_N, DCM/dioxane 1:1, 0 °C → rt, 48 h, 82%; c) TFA/DCM 1:2, rt, 1 h followed by chromatographic separation of isomers, 43%.

Peptide building blocks were prepared starting from the fully protected desmuramyl peptide, Boc-ʟ-Ala-ᴅ-isoGlnOBn. For the synthesis of derivatives lacking the adamantane moiety, dipeptide **1** with benzyl protection on the C-terminus was obtained after Boc-deprotection as previously reported (Scheme 1) [31]. Peptide **1** was also used for the synthesis of adamantly-containing desmuramyl tripeptide **3**.

**Scheme 2:**
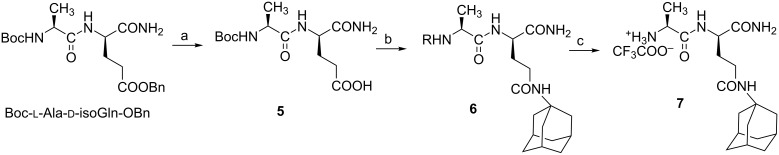
Synthesis of C-modified desmuramyl peptides. Reagents and conditions: a) H_2_, 10% Pd/C, MeOH, 38 psi, rt, 24 h, 96%; b) adamant-1-ylamine hydrochloride, EDC·HCl, HOBt·H_2_O, Et_3_N, DCM/dioxane 1:1, 0 °C → rt, 48 h, 60%; c) TFA/DCM 1:2, rt, 1 h, quantitative.

Compound **1** was coupled with previously prepared racemic Boc-protected (adamant-1-yl)glycine [32] **2** using the carbodiimide EDC/HOBt method [21]. The obtained mixture of BocAdGly-ʟ-Ala-ᴅ-isoGlnOBn diastereoisomers was treated with trifluoroacetic acid in order to remove the Boc protecting group while the diastereoisomer **4** with ᴅ-ʟ-ᴅ amino acid sequence was separated from the isomer mixture using silica gel column chromatography and CHCl_3_/MeOH 1:1 as eluent. The spectral data of the isolated isomer **4** were compared to the published ᴅ-AdGly-ʟ-Ala-ᴅ-isoGln prepared from tripeptide *tert*-butyl ester [21]. Desmuramyl peptides **1** and **4** were further used for condensation reactions with hydroxyisobutyryl and glycolyl mannosides. The synthesis of desmuramyl peptide **7** with an adamantane moiety bound at C-terminus is presented in Scheme 2.

**Scheme 3:**
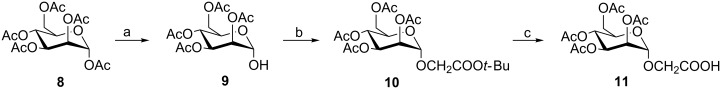
Synthesis of the mannose precursor. Reagents and conditions: a) Zn(OAc)_2_·H_2_O, abs. MeOH, rt, 20 h, 60%; b) BrCH_2_COOC(CH_3_)_3_, K_2_CO_3_, dry DMF, rt, 2 h, 81%; c) TFA, dry DCM, rt, 1,5 h, 72%.

After hydrogenolysis of the starting dipeptide, condensation of free carboxyl group with adamant-1-ylamine hydrochloride was performed. Boc deprotection of obtained compound **6** gave the trifluoroacetic salt of peptide **7** which was used in the synthesis of mannoconjugates. The mannose precursor containing the glycolyl linker **11** was prepared in a three-step procedure shown in Scheme 3.

**Scheme 4:**
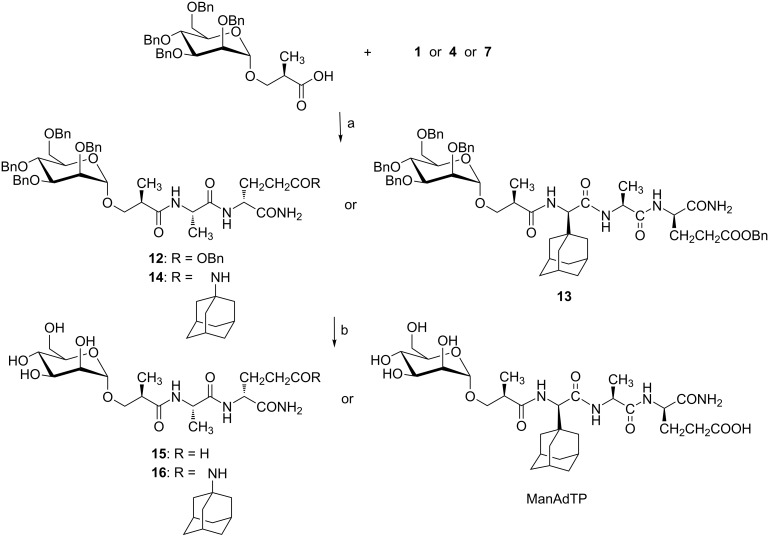
Synthesis of mannosylated peptides with hydroxyisobutyryl linker. Reagents and conditions: a) EDC·HCl, HOBt·H_2_O, Et_3_N, DCM/dioxane 1:1, 0 °C → rt, 48 h, 52–90%; (b) H_2_, 10% Pd/C, 48 h, rt, 83–92%.

**Scheme 5:**
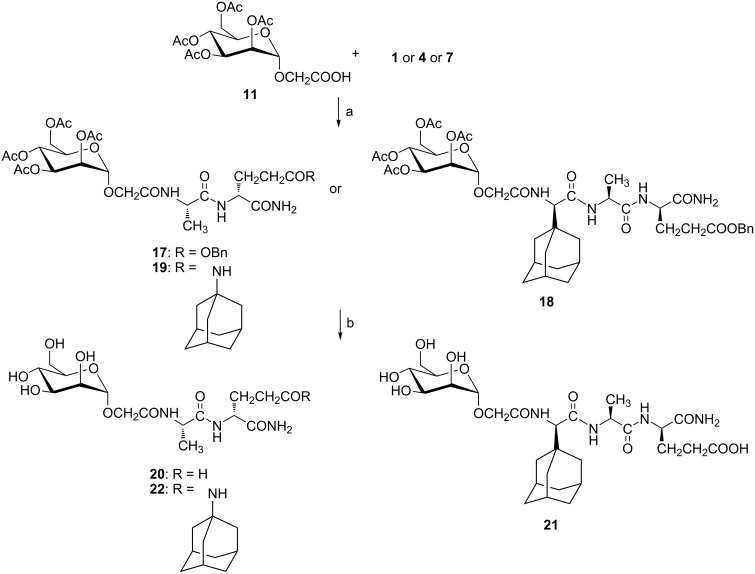
Synthesis of mannosylated peptides with glycolyl linker. Reagents and conditions: a) EDC·HCl, HOBt·H_2_O, Et_3_N, DCM/DMF 1:1, 0 °C → rt, 72 h, 26–67%; b) NaOMe/MeOH, rt, 1 h, 59–89%.

The stereoselective α-anomeric deacetylation of peracetylated mannose **8** was performed using the mild acidic catalyst, zinc acetate hydrate [33]. The reaction progress was monitored by thin-layer chromatography. Within the first 20 hours compound **9** was the only product and further progress resulted in gradual removal of the remaining acetate groups. The S_N_2 substitution of bromine from *tert*-butyl bromoacetate with 2,3,4,6-tetra-*O*-acetyl-α-ᴅ-mannopyranose (**9**) in the presence of potassium carbonate followed. Chemoselective removal of the *tert*-butyl ester group from compound **10** resulted with *O*-mannoside **11** with a free carboxy group available for coupling of the peptide moieties. The *tert-*butyl deprotection was accomplished using a selective reagent, trifluoroacetic acid (TFA). Side products derived from deacetylation of compound **10** have not been detected. The synthesis of benzyl-protected α-mannoside containing a (*R*)-hydroxyisobutyryl linker was previously described [34]. Condensations of peptides **1**, **4** and **7** with carboxy-functionalized mannosides containing a (*R*)-hydroxyisobutyryl and glycolyl linker are shown in Scheme 4 and Scheme 5, respectively.

For the amide bond formation between mannose and peptide part, an optimized EDC/HOBt method was used in each case. Synthesized compounds represent a small series of mannosylated desmuramyl peptides designed in a way that the introduced structural differences could answer two key questions: (i) Will the glycolyl linker in this class of compounds amplify immunostimulatory activity similar to the N-glycolyl derivative of MDP?, (ii) which relative position of the adamantane group in the mannosylated desmuramyl peptides causes the greatest increase of the adjuvant activity?

### Testing of immunostimulating activity

**Figure 4:**
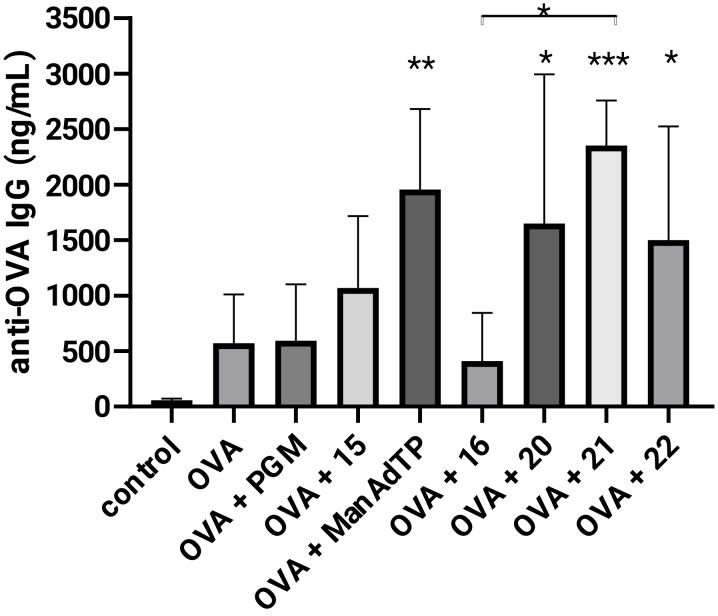
The effect of mannosyl desmuramyl peptides on production of anti-OVA IgG in BALB/c mice immunized with OVA as an antigen. Bar graphs represent average values from individual mice from each group (*n* = 5). *p < 0.05, **p < 0.01 and ***p < 0.001 denote the statistical significance in comparison to the control group or groups connected with a line.

The adjuvant activity was estimated by the immunostimulatory effect on secondary humoral response to the well-established model antigen ovalbumin (OVA) in BALB/c mice (Figure 4) according to previously described in vivo studies [11,35]. Anti-OVA IgG, anti-OVA IgG1 and anti-OVA IgG2a were determined in the mice sera after supplementing the mice with the second booster. The comparison of induced anti-OVA IgG levels was carried out quantitatively and the subclasses of IgG, IgG1 and IgG2a, as indicators of Th1 or Th2 type of immune response, were also determined. The adjuvant activity of synthesized compounds was evaluated in comparison to the mannosylated adamantyl tripeptide ManAdTP and PGM. PGM was used as reference adjuvant in previously published studies since PGM and MDP have similar immunological properties: both stimulate the Th2-biased immune response specific for the OVA antigen [11,35].

In general, when compared to the group treated with no adjuvant (OVA alone), enhancement in total anti-OVA IgG antibody production was observed in all groups except in group that received **16** (Figure 4). High levels of IgG antibody present even in the OVA treated group, led to a relatively weak stimulation of the total antibody production in the PGM-injected group. ManAdTP elicited a better immune response than OVA alone and the PGM-injected group. These results are in very good agreement with previous research where stimulation of anti-OVA IgG antibody production by ManAdTP and parent non-mannosylated AdTP were investigated in the NIH/OlaHsd mouse model in comparison to PGM [36]. In BALB/c mice used in this study, enhancement in the total anti-OVA IgG antibody production by ManAdTP is statistically significant (*p* < 0.01). A statistically significant boost in antibody responses to the OVA antigen was observed in all groups immunized with compounds containing a glycolyl linker (*p* < 0.05 and and *p* < 0.001) in comparison to the control group. Immunization with compound **21** which has an adamantly tripeptide moiety attached to mannose through the glycolyl linker led to the highest and statistically most significant increase in the specific IgG response (*p* < 0.001). Additionally, amplification in the total IgG antibody production was observed in all groups immunized with mannosylated desmuramyl peptides containing a glycolyl linker **20**–**22** relatively to analogues **15**, ManAdTP and **16**. The results directly indicate that the introduction of the glycolyl linker plays a significant role in stimulation of the immune response in this class of adjuvants. Similarly, *N*-glycolyl muramyl peptides obtained by the oxidation of the *N*-acetyl group of MDP induce significantly a higher activation of NOD2 than MDP [29–30]. Glycopeptide **21** was identified as the most potent adjuvant in this experiment and in this class of adjuvants, so far. Introduction of the lipophilic moiety positively influences the adjuvant properties of MDP derivatives and analogues [12]. In both, hydroxyisobutyryl and glycolyl derivatives, introduction of the bulky and lipophilic adamantane showed to be suitable for the immunostimulatory activity. The adjuvant activity changes in respect to the position of adamant-1-yl moiety in the peptide part. Anti-OVA IgG antibody stimulation was higher in the groups immunized with ManAdTP and **21** in respect to groups treated with **16** and **22**, respectively. This leads to the conclusion that the most suitable position of adamantane in this class of compounds is at the peptide N-terminus. Adamantane can act as membrane anchor for mannose structures and thus be exposed on liposome surfaces and as such used in targeted drug delivery [37]. It can be also incorporated into a β-cyclodextrine cavity, a powerful supramolecular nanoparticle carrier for targeted drug delivery [38]. Previous research suggested a design of mannosylated desmuramyl peptides with adamantane at the C-terminus in order to facilitate the incorporation into the hydrophobic layer of the cavity because of the minor steric hindrance of the mannose and peptide part during the inclusion process of the adamantane [37,39].

**Figure 5:**
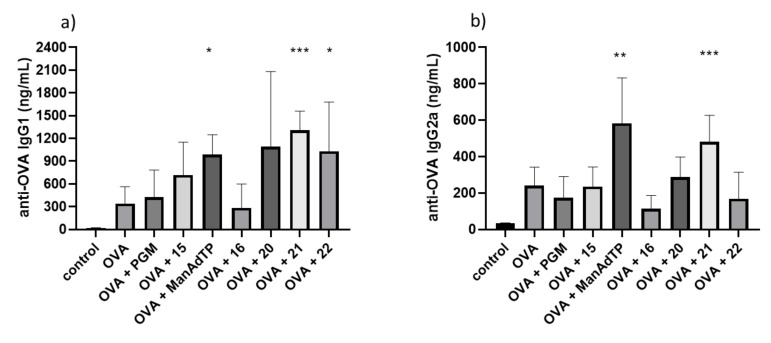
The effect of mannosylated desmuramyl peptides on production of anti-OVA IgG subtypes, anti-OVA IgG1 (a) and anti-OVA IgG2a (b), respectively, in BALB/c mice immunized with OVA as an antigen. Bar graphs represent average values from individual mice from each group (*n* = 5). *p < 0.05 and ***p < 0.001 denote statistical significance in comparison to the control group.

It is well known that vaccine adjuvants can enhance or modulate the Th1/Th2-bias of an induced immune response. Interferon-γ (as a Th1 cytokine) and IL-4 (as a Th2 cytokine) induce isotype switching to IgG2a and IgG1, respectively. Therefore, isotype profile of antigen specific anti-OVA IgG antibodies, IgG1 and IgG2a, is usually measured as a marker of the Th1 and Th2 type immune response bias [40–41]. In this study, the type of generated immune response was indirectly estimated by quantification of OVA-specific IgG1 (for activation of Th2 type) and IgG2a (for activation of Th1 type) and calculation of the respective IgG1/IgG2a ratio. When the amount of anti-OVA IgG1 antibodies was measured (Figure 5a), it was observed that in all groups high levels of IgG1 antibody was present and the highest response, which was also statistically significant (*p* < 0.001), was given by compound **21**. A slight suppression was noticed only in the production of anti-OVA IgG1 antibodies when compound **16** was administered.

**Figure 6:**
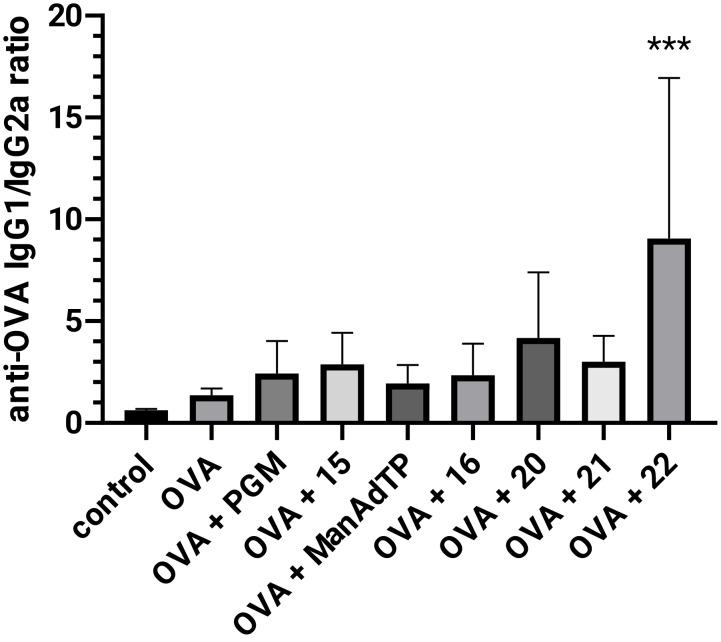
The ratio of anti-OVA IgG1 and anti-OVA IgG2a (IgG1/IgG2a) in BALB/c mice. For each mouse serum IgG1/IgG2a was calculated and the result for each experimental group (*n* = 5) is presented as average ± standard deviation (SD). ***p < 0.001 denote statistical significance in comparison to the control group.

A statistically significant enhancement in anti-OVA IgG2a production (Figure 5b) was observed in groups immunized with mannosylated adamantyl-tripeptides, ManAdTP and **21**. The type of immune response was indirectly determined by quantification of IgG1 (for activation of Th2-type) and IgG2a (for activation of Th1-type) antibody for each serum (obtained after the second booster) and calculation of the IgG1/IgG2a ratio (Figure 6).

From the IgG1/IgG2a ratio it is evident that all groups treated with tested adjuvants have higher values than the group treated with OVA alone, indicating the slight shift toward more pronounced Th2 type of immune response. Compound **22** significantly switches immune response toward pronounced Th2 type, due to the predominant appearance of IgG1 antibodies. MDP and PGM dominantly induce the IgG1 antibody production and stimulate the Th2-polarized immune response as well [11,42]. It is well known that muropeptides act as NOD2 agonists and induce a predominant Th2-biased response [43]. NOD2 agonists have the ability to act synergistically and augment the adjuvant activity of TLR ligands [10,44–46]. Furthermore, a combination of PRR ligands, such as NLR/TLR, induce Th1-polarized response. This NLR/TLR crosstalk is essential for modulation of innate and adaptive immune responses and leads to development of new approaches for the design of novel vaccines. Application of multi-PRR activation approaches can increase the immunity significantly [47]. Another example of dual adjuvant system is represented by the activation of dendritic cells via combined macrophage-inducible CLR and TLR ligands [48]. Mannose receptors are one group in the CLRs family which exist as soluble and transmembrane receptors [4]. Like TLRs they initiate innate immune responses and activate acquired immunity. Mannose structures on the other hand, are one of the glycan structures that build up tumor antigens and regulate the immune reaction by specific binding to CLRs. Therefore, compounds with expressed CRL agonist or antagonist properties could also be considered as potential agents for cancer immunotherapy [49–50]. Mannosylated liposomes with incorporated MDP have proved to be effective carriers for target inhibition of liver metastasis [51]. Therefore, the presented mannosylated desmuramyl peptides with incorporated adamantane will be further explored in order to get a better insight into possible PRR crosstalk. Namely, inclusion of adamantane into carriers such as liposomes can additionally affect the Th1/Th2 switch of the immune response [52]. The presented results demonstrate the great immunostimulating potential of glycolyl-modified desmuramyl peptides. Peptidoglycan fragments with mycobacterial structural features, such as synthesized compounds **20**–**22**, could efficiently link innate and adaptive immunity, similarly as *N*-glycolyl MDP enhances the innate immune response and T cell-mediated immunity [53]. NOD2-activation and interaction with CLR should be further explored, as well as a potential for synergistic multi-PRR activation.

## Conclusion

A series of novel mannosylated desmuramyl peptides were prepared and characterized. In their structures, all glycopeptides comprised of mannose and the key pharmacophore – desmuramyl peptide. These moieties are connected through a glycolyl or hydroxyisobutyryl linker and additionally modified on N/C terminus with an adamantane subunit. The immunostimulating activities of tested compounds were compared to hit compounds ManAdTP and PGM. In in vivo experiments, all mannopeptides with a glycolyl linker exhibited higher adjuvant activity than analogues with a hydroxyisobutyryl linker indicating that the introduction of the glycolyl moiety plays a significant role in the stimulation of the immune response. In particular, compound **21** was identified, so far, as the most potent adjuvant in this class of mannosylated desmuramyl peptides. It should be also noted that the compound **21** is stable, non-pyrogenic and water-soluble what makes it potentially applicable as an adjuvant for vaccines.

## Supporting Information

File 1 Experimental and characterization data.
