# Supplementary material for: Design, synthesis and biological evaluation of immunostimulating mannosylated desmuramyl peptides
Source: Beilstein J Org Chem. 2019 Jul 29;15:1805–14. doi: 10.3762/bjoc.15.174 (PMC6693374; doi:10.3762/bjoc.15.174)
Supplement: File 1 — Experimental and characterization data. [file Beilstein_J_Org_Chem-15-1805-s001.pdf]

BEILSTEIN JOURNAL OF ORGANIC CHEMISTRY

Supporting Information

for

Design, synthesis and biological evaluation of immunostimulating mannosylated desmuramyl peptides

Rosana Ribić, Ranko Stojković, Lidija Milković, Mariastefania Antica, Marko Cigler and Srđanka Tomić

Beilstein J. Org. Chem. **2019,** *15,* 1805–1814. doi:10.3762/bjoc.15.174

Experimental and characterization data

License and Terms: This is a supporting information file under the terms of the Creative Commons Attribution License (http://creativecommons.org/licenses/by/4.0). Please note that the reuse, redistribution and reproduction in particular requires that the authors and source are credited. The license is subject to the *Beilstein Journal of Organic Chemistry* terms and conditions: (https://www.beilstein-journals.org/bjoc)

Materials and methods — ***S3***

General procedure for coupling — ***S3***

General procedure for peptide Boc deprotection — ***S4***

General procedure for debenzylation — ***S4***

General procedure for deacetylation of mann oconjugates — ***S4***

Characterization data — ***S5–S19***

Experiment in vivo — ***S20***

Measurements of antibody — ***S20***

Statistics — ***S21***

Materials and methods

Boc-L-Ala-D-isoGln-OBn was obtained from Bachem (Switzerland) and other reagents and solvents for the synthesis of compounds were obtained from Sigma-Aldrich Corp. Organic solvents were further purified and/or dried using standard methods. Thin layer chromatography (TLC) was performed on Fluka silica gel (60 F_{254}) plates (0.25 mm). Visualization was achieved using UV light at 254 nm and ninhydrine. Column chromatography was performed on Merck silica gel 60 (size 70–230 mesh ASTM). The purity of compounds was analyzed by HPLC (Shimadzu HPLC) with photodiode array detector (SPD-M20A) and autosampler (SIL-20ACTH). All tested compounds have purity over 95% at 214 nm. Analyses were run at Shim-pack GIST C-18 column, 250 mm × 4.6 mm, 5 µm, with flow rate of 1.0 mL/min at room temperature. The gradient solvent system used was made of acetonitrile and water. The percentage of acetonitrile at 0, 5 and 8 min was 10, 30 and 10, respectively. Acetonitrile and water were HPLC quality. Mass spectra were recorded on Agilent 6410 MS instrument. High-resolution mass spectra (HRMS) were obtained by matrix-assisted laser desorption/ionization time-of-flight MALDI–TOF/TOF mass spectrometer (4800 Plus MALDI TOF/TOF analyzer; Applied Biosystems Inc.). Optical rotations were measured at room temperature using the Schmidt + Haensch Polartronic NH8 instrument. ^{1}H and ^{13}C NMR spectra of all precursors were recorded on Bruker AV-600 spectrometer. All NMR experiments were performed at 298 K. Chemical shifts were referenced with respect to tetramethylsilane (TMS).

General procedure for coupling

Compound with the free carboxylic group was dissolved in anhydrous CH_2Cl_2 and cooled at 0 °C. EDC hydrochloride (1.2 equiv) and HOBt hydrate (1 equiv) were added to the solution and the mixture was stirred for 0.5 h at 0 °C. Then, this mixture was added to the solution of

compound with the free amine group (1 equiv), in anhydrous 1,4-dioxane, followed by triethylamine (2 equiv). The reaction mixture was stirred for 1 h at 0 °C and then for 48 h at room temperature. The mixture was diluted with AcOEt and washed with water. The organic layer was dried over anhydrous $MgSO_4$ and, after filtration, concentrated in vacuo. The residue was purified via column chromatography on silica gel ($CHCl_3$/MeOH 9:1) to yield (glyco)peptide.

General procedure for peptide Boc-deprotection

The Boc-protected peptide was suspended in anhydrous CH_2Cl_2 and TFA (CH_2Cl_2/TFA 2:1) was added. The reaction mixture was stirred at room temperature for 1 h (until the disappearance of the Boc-protected peptide was confirmed via TLC) and then concentrated in vacuo. The residue was purified by flash chromatography on silica gel to yield peptide TFA salt as viscous oil.

General procedure for debenzylation

Benzyl protected peptide was dissolved in MeOH, 10% Pd/C was added and it was subjected to hydrogen atmosphere under 35 psi at room temperature for 24–48 h. The catalyst was then filtered, the solvent removed in vacuo and the residue purified by column chromatography on silica gel.

General procedure for deacetylation of manno conjugates

To a solution of an appropriate acetylated mannosides in dry MeOH, 25% solution of sodium methoxide in dry methanol (5 equiv) was added. The reaction mixture was stirred for approximately 1 h at room temperature and monitored by TLC. The mixture was then purified

without previous evaporation of the solvent by column chromatography on silica gel giving deacetylated products.

tert-Butoxycarbonyl-L-alanyl-D-isoglutamine (**5**)

Starting from benzyl ester of N-Boc-L-Ala-D-*iso*Gln (200 mg, 0.49 mmol) product **5** was obtained as colourless powder (153 mg, 96%): R_f=0.39 ($CHCl_3$/MeOH 2:1); ^{1}H NMR (300 MHz, CD_3OD): δ=4,36 (dd, J=4.26, 10.25 Hz, 1H, CH isoGln), 4.02 (q, J=7.15 Hz, 1H, CH Ala), 2.38 (pt, J=7.80, 7.30 Hz, 2H, γ-CH_2 isoGln), 2.20 (m, 1H, β-CH isoGln), 1.90 (m, 1H, β-CH isoGln), 1.43 (s, 9H, Boc), 1.30 (d, J=7.16 Hz, 3H, CH_3 Ala) ppm; ^{13}C NMR (75 MHz, CD_3OD) δ=176.50, 176.47, 176.42, 176.40 (C=O), 80.79 (C Boc), 53.70 (CH isoGln), 52.09 (CH Ala), 31.29 (γ-CH_2 isoGln), 28.74 (CH_3 Boc), 28.15 (β-CH_2 isoGln), 17.73 (CH_3 Ala) ppm; ESI-MS m/z $[M+Na]^+$ calcd for $C_{13}H_{22}N_3O_6$: 340.1, found: 340.1.

N-(Adamantan-1-yl)-(tert-butoxycarbonyl)-L-alanyl-D-isoglutamine 5-amide (**6**)

Condensation of peptide **5** (340 mg, 1.08 mmol) and 1-aminoadamantane hydrochloride (222 mg, 1.18 mmol) gave the product **6** as colourless powder (288 mg, 60%): R_f=0.69 ($CHCl_3$/MeOH 5:1); ^{1}H NMR (300 MHz, CD_3OD) δ=4.30 (dd, J=3.46, 10.78 Hz, 1H, CH isoGln), 4.00 (q, J=7.16 Hz, 1H, CH Ala), 2.23-2.12 (m, 3H, isoGln), 2.02 (bs, 9H, CH Ad, CH_2 Ad), 1.91-1.79 (m, 1H, β-CH isoGln), 1.71 (bs, 6H, CH_2 Ad), 1.44 (s, 9H, Boc), 1.32 (d, J=7.16 Hz, 3H, CH_3 Ala) ppm; ^{13}C NMR (75 MHz, CD_3OD) δ=176.44, 176.20, 174.11, 174.03 (C=O), 80.82 (C Boc), 53.60 (CH isoGln), 52.94 (C Ad), 52.30 (CH Ala), 42.32 (CH_2 Ad), 37.52 (CH_2 Ad), 34.11 (γ-CH_2 isoGln), 30.93 (CH Ad), 29.06 (β-CH_2 isoGln), 28.72 (CH_3 Boc), 17,65 (CH_3 Ala) ppm; MS (ESI) m/z $[M+H]^+$ calcd for $C_{23}H_{38}N_4O_5$: 451.6, found: 451.3.

N-(Adamantan-1-y)-L-alanyl-D-isoglutamine 5-amide trifluoroacetate (**7**)

Compound **7** was obtained in quantitative yield starting from compound **6** as colourless powder (103 mg, >99%): R_f=0.16 ($CHCl_3$/MeOH 2:1); ^{1}H NMR (300 MHz, CD_3OD) δ=4.33 (dd, J=5.10, 8.88 Hz, 1H, CH *iso*Gln), 3.97 (q, J=7.06 Hz, 1H, CH Ala), 2.23 (pt, J=7.10; 7.47 Hz, 2H, γ-CH_2 isoGln), 2.14-2.08 (m, 1H, β-CH isoGln), 2.06-2.02 (m, 9H, CH Ad, CH_2 Ad), 1.96-1.82 (m, 1H, β-CH isoGln), 1.71 (bs, 6H, CH_2 Ad), 1.53 (d, J=7.07 Hz, 3H, CH_3 Ala) ppm; ^{13}C NMR (75 MHz, CD_3OD) δ/ppm 176.07, 173.87, 171.07 (C=O), 54.09 (CH isoGln), 52.86 (C Ad), 50.34 (CH Ala), 42.31 (CH_2 Ad), 37.48 (CH_2 Ad), 33.79 (γ-CH_2 isoGln), 30.90 (CH Ad), 29.18 (β-CH_2 isoGln), 17.63 (CH_3 Ala) ppm; MS (ESI) *m/z* $[M+H]^+$ calcd for $C_{18}H_{30}N_4O_3$: 351.5, found: 351.3.

tert-Butyl-2-(2,3,4,6-tetra-O-acetyl-α-D-mannopyranosyloxy)acetate (**10**)

Compound **9** (0.372 g, 1.068 mmol) was dissolved in dry *N,N*-dimethylformamide (5 mL) and then *tert*-butylbromoacetate (0,236 mL) and potassium carbonate (0.738 g) were added. Reaction mixture was stirred for 2 h at room temperature and afterword filtered. Beaker was washed with diethyl-eter (20 mL). Mixture was extracted three times with water (15 mL). Organic layer was dried over anhydrous sodium sulfate. After filtration, solvent was removed in vacuo. Product was purified by column chromatography on silica gel ($CHCl_3$/CH_3CN 3:1) and of compound **10** was obtained as yellow viscous oil (398 mg, 81%): R_f=0.64 ($CHCl_3$/CH_3CN 3:1); $[\alpha]_D^{25}$=+48.5 (c=1.2 in $CHCl_3$); ^{1}H NMR (300 MHz, $CDCl_3$) δ=5.41-5.28 (m, 3H, H-2, H-3, H-4), 4.96 (d, $J_{1,2}$=1.0 Hz, 1H, H-1), 4.30 (dd, $J_{5,6}$=4.8, $J_{6a,6b}$=12.2 Hz, 1H, H-6b), 4.20 – 4.02 (m, 4H, H-5, H-6a, OCH_2), 2.26 (s, 3H, CH_3), 2.11 (s, 3H, CH_3), 2.04 (s, 3H, CH_3), 1.99 (s, 3H, CH_3), 1.48 (s, 9H, CH_3 *t*-Bu) ppm; ^{13}C NMR (75 MHz, $CDCl_3$) δ=170.62, 169.78, 169.75, 169.69, 168.17 (C=O), 97.70 (C1), 82.30 (C *t*-Bu), 69.32, 69.03, 68.87, 65.92 (C2, C3, C4, C5), 64.89, 62.29 (C6, OCH_2), 28.03 (CH_3 *t*-Bu), 20.84, 20.73, 20.68, 20.64 (CH_3 Ac); MS (ESI) *m/z* $[M+Na]^+$ calcd for $C_{20}H_{30}O_{12}$: 485.1, found: 485.2.

2-(2,3,4,6-Tetra-O-acetyl-α-D-mannopyranosyloxy)acetic acid (**11**)

Compound **10** (1.11 g, 2.4 mmol) was dissolved in dry dichloromethane (5 mL) and trifluoroacetic acid (2 mL) was added. Reaction mixture was stirred for 3 h and then diluted with dichloromethane (30 mL). Mixture was washed with water (20 mL) and water layer was extracted additionally with dichloromethane (20 mL). Organic layers were dried over anhydrous sodium sulfate and after filtration organic solvent was removed in vacuo. Product was purified by column chromatography on silica gel ($CHCl_3$/MeOH 15:1) and compound **11** was obtained as yellow viscous oil (856 mg, 72%):

R_f=0.62 ($CHCl_3$/MeOH 15:1); $[\alpha]_D^{25}$=+29.0 (c=1.0 in MeOH); ^{1}H NMR (300 MHz, CD_3OD) δ=5.35-5.25 (m, 3H, H-2, H-3, H-4), 4.95 (d, $J_{1,2}$ = 0.9 Hz, 1H, H-1), 4.58 (s, <1H, OH); 4.32-4.16 (m, 4H, H-5, H-6a, OCH_2), 4.11 (dd, $J_{5,6}$=1.7, $J_{6a,6b}$ =11.5 Hz, 1H, H-6b), 2,14 (s, 3H, CH_3), 2.06 (s, 3H, CH_3), 2.04 (s, 3H, CH_3), 1.96 (s, 3H, CH_3) ppm; ^{13}C NMR (75 MHz, CD_3OD) δ=172.45, 172.57, 171.50 (C=O), 99.17 (C1), 70.69, 70.49, 70.42, 67.16 (C2, C3, C4, C5), 63.50 (C6, OCH_2), 20.68, 20.65, 20.60 (CH_3) ppm; MS (ESI) m/z $[M+Na]^+$ calcd for $C_{16}H_{22}O_{12}$ 429.0; found 429.2.

*[(R)-3-(2,3,4,6-Tetra-*O*-benzyl-α-D-mannopyranosyloxy)-2-methylpropanoyl]-L-alanyl-D-isoglutamine benzyl ester* (**12**)

Starting from compound **1** (259 mg, 0.35 mmol) pure product **12** was obtained in the form of viscous oil (166 mg, 52%): R_f = 0.52 ($CHCl_3$/MeOH 9:1); ^{1}H-NMR (300 MHz, $CDCl_3$) δ=7.35-7.25 (*m*, 25H, H_{arom}), 5.58 (*s*, 1H, H-1), 5.11 (d, J=12.3 Hz, 1H, CH Bn), 5.09 (d, J=12.3 Hz, 1H, CH Bn), 4.86-4.47 (m, 6H, CH_2 Bn), 4.37 (dd, J = 5.1; 8.3 Hz, 1H, CH isoGln), 4.25 (q, J=6.8 Hz, 1H, CH Ala), 3.85-3.64 (m, 7H, H-2, H-3, H-4, H-5, H-6, CH linker), 3.53 (dd, J=4.4, J=10.4 Hz, 1H, CH linker), 2.69-2.63 (m, 1H, CH linker), 2.55-2.50 (m, 1H, γ-CH isoGln), 2.43-2.38 (m, 1H, γ-CH isoGln), 2.20-2.15 (m, 1H, β-CH isoGln),

1,99-1,93 (m, 1H, β-CH isoGln), 1.21 (d, *J* = 7.0 Hz, 3H, CH_3 Ala), 0.97 (d, *J* = 7.0 Hz, 3H, CH_3 linker); ^{13}C NMR ($CDCl_3$, 150 MHz) δ=175.41, 173.35, 173.24, 172.56 (C=O), 138.42, 138.25, 138.22, 137.77, 135.61 (C_{arom}), 128.58-127.53 (CH_{arom}), 99,94 (C-1), 79.93, 75.11, 74.98, 72.04 (C2, C3, C4, C5), 74,81, 73.66, 72.78, 72.23, 71.88, 69.92 (C-6, CH_2 Bn, CH_2 linker), 52.36 (CH isoGln), 49.40 (CH Ala), 41.03 (CH linker), 30.59 (γ-CH_2 isoGln), 26.76 (β-CH_2 isoGln), 17.22 (CH_3 Ala), 14.00 (CH_3 linker); MS (ESI) *m/z*: $[M+H]^+$ calcd for $C_{53}H_{61}N_3O_{11}$ 916.4, found 916.6.

*[(*R*)-3-(2,3,4,6-Tetra-*O*-benzyl-α-D-mannopyranosyloxy)-2-methylpropanoyl]-D-(adamant-1-yl)glycyl-L-alanyl-D-isoglutamine benzyl ester* (**13**)

Starting from compound **4** (275 mg, 0.35 mmol) pure product **13** was obtained in the form of viscous oil (246 mg, 64%): R_f=0.36 ($CHCl_3$/MeOH 12:1); ^{1}H NMR (600 MHz, $CDCl_3$) δ=7.34-7.23 (m, 20 H, H_{arom}), 4.91 (d, *J*=1.7 Hz, 1H, H-1), 5.07 (d, 12.3 Hz, 2H, CH_2 Bn), 4.84-4.53 (m, 6H CH_2 Bn), 4.53-4.48 (m, 2H, CH *iso*Gln, CH Ala), 3.94-3.71 (m, 6H, H-4, H-3, H-5, H-2, H-6), 3.65-3.57 (m, 2H, CH_2 linker), 2.71-2.67 (m, 1H, CH linker), 2.46-2.33 (m, 2H, γ-CH *iso*Gln), 2.17-2.11 (m, 1H, β-CH *iso*Gln), 1.93-1.87 (m, 1H, β-CH *iso*Gln), 1.85-1.47 (m, 15H, CH Ad), 1.27 (d, *J*=7.0 Hz, 3H, CH_3 Ala), 0.99 (d, *J*=7.0 Hz, 3H, CH_3 linker) ppm; ^{13}C NMR (150 MHz, $CDCl_3$) δ=174.84 173.24, 172.73, 172.18, 169.72 (C=O), 138.54, 138.34, 139.31, 138.23, 135.74 (C_{arom}), 128.54-127.45 (CH_{arom}), 98.90 (C-1), 80.40, 75.14, 75.06, 72.18 (C-2, C-3, C-4, C-5), 75.04, 73.51, 72.83, 72.21 70.49 (C-6, CH_2 Bn, CH_2 linker), 61.66 (CH AdGly), 51.79 (CH *iso*Gln), 49.04 (CH Ala), 41.02 (CH linker), 38.78, 36.69, 36.60 (CH_2 Ad), 30.43 (γ-CH_2 isoGln), 28.28 (β-CH_2 *iso*Gln), 28.18 (CH Ad), 18.96 (CH_3 Ala), 14.13 (CH_3 linker) ppm; HRMS calcd for $[C_{65}H_{78}N_4O_{12}+H]^+$ 1107.5695, found: 1107.5675.

*N-(Adamantan-1-yl)-[(*R*)-3-(2,3,4,6-tetra-*O*-benzyl-α-D-mannopyranosyloxy)-2-methylpropanoyl]-L-alanyl-D-isoglutamine 5-amide* (**14**)

Starting from compound **7** (120 mg, 0.24 mmol) pure product **14** was obtained in the form of viscous oil (205 mg, 90%): R_f=0.85 ($CHCl_3$/MeOH 5:1); ^{1}H NMR (600 MHz, CD_3OD) δ=7.39-7.16 (m, 20 H, H_{arom}), 4.88 (d, *J*=1.5 Hz, 1H, H-1), 4.80 (s, 1H, NH), 4.68 (dd, *J*=8.6; 12.1 Hz, 2H, CH_2 Bn), 4.63-4.48 (m, 6H, CH_2 Bn), 4.28 (dd, *J*=4.1; 9.3 Hz, 1H, CH isoGln), 4.25 (q, *J*=7.2 Hz, 1H, CH Ala), 3.89 (t, *J*=9.2 Hz, 1H, H-4), 3.84 (dd, *J*=3.0; 9.1 Hz, 1H, H-3), 3.81 (pt, *J*=1.9; 2,9; 1H, H-5), 3.74-3.64 (m, 4H, H-2, H-6, CH linker), 3.57 (t, *J*=9.3 Hz, 1H, CH linker), 2.75-2.68 (m, 1H, CH linker), 2.23-2.09 (m, 3H, γ-CH_2 isoGln, β-CH isoGln), 2.01 (bs, 9H, CH_2 Ad), 1.89-1.82 (m, 1H, β-CH isoGln), 1.69 (bs, 6H, CH_2 Ad), 1.31 (d, *J*=7.3 Hz, 3H, CH_3 Ala), 1.06 (d, *J*=7.0 Hz, 3H, CH_3 linker) ppm; ^{13}C NMR (150 MHz, CD_3OD) δ=177.57, 176.38, 175.23, 174.18 (C=O), 139.84, 139.71, 139.59 (C_{arom}), 129.45-128.70 (CH_{arom}), 99.68 (C-1), 81.27, 76.05, 76.01, 73.36 (C-2, C-3, C-4, C-5), 74.52, 73.70, 73.00, 71.35, 70.54 C-6, CH_2 Bn, CH_2 linker), 53.84 (CH isoGln), 53.00 (C Ad), 51.09 (CH Ala), 42.36 (CH_2 Ad), 41.89 (CH linker), 37.55 (CH_2 Ad), 34.12 (γ-CH_2 isoGln), 30.95 (CH Ad), 29.08 (CH_2 isoGln), 17.86 (CH_3 Ala), 14.39 (CH_3 linker) ppm; HRMS calcd for $[C_{50}H_{70}N_4O_{10}+H]^+$ 959.5171, found: 959.5164.

[(R)-3-(α-D-Mannopyranosyloxy)-2-methylpropanoyl]-L-alanyl-D-isoglutamine (**15**)

Starting from compound **12** (70 mg, 0.06 mmol) pure product **15** was obtained in the form of viscous oil (27 mg, 92%): R_f = 0.57 ($CHCl_3$/MeOH 1:1); ^{1}H-NMR (300 MHz, CD_3OD) δ=4.95 (s, 1H, H-1), 4.45-4.36 (m, 3H, CH Ala, CH isoGln, 6a), 4.30-4.11 (m, H-2, H-3, H-4, H-5, H-6b, CH_2 linker), 2.47-2.41 (m, 3H, CH linker, γ-CH_2 isoGln), 2.29-2.18 (m, 1H, β -CH isoGln), 1.98-1.89 (m, 1H, β-CH isoGln), 1.45 (d, *J*=7.1 Hz, 3H, CH_3 Ala), 1.43 (d, *J*=7.2 Hz, 3H, CH_3 linker) ppm; ^{13}C NMR ($CDCl_3$, 75 MHz) δ=177.64, 176.25, 175.53 (C=O), 102,24 (C-1), 74.83, 72.62, 72.10, 68.67 (C-2, C-3, C-4, C-5), 71.14, 63.00 (C-6, CH_2 linker), 53.62

(CH isoGln), 50.97 (CH Ala), 41.92 (CH linker), 31.17 (γ-CH_2 isoGln), 27.98 (β-CH_2 isoGln), 17.73 (CH_3 Ala), 14.41 (CH_3 linker); HPLC: t_R=3.15 min; ESI-MS *m/z* $[M+H]^+$ calcd for $C_{18}H_{31}N_3O_{11}$: 488.1, found 488.2.

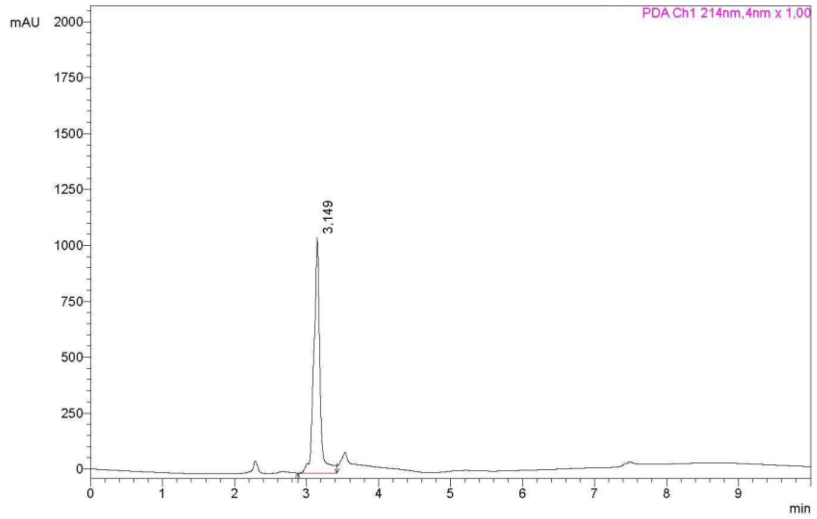

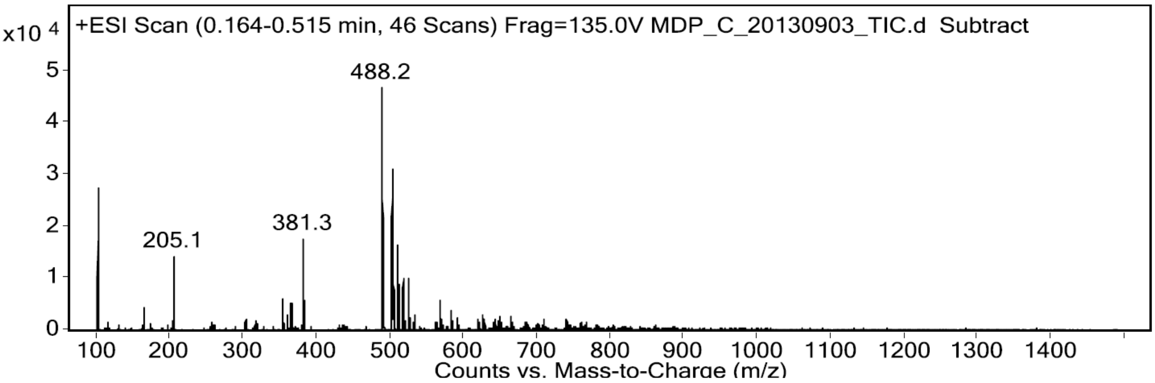

MALDI TOF/TOF mass spectrometry using positive ionization (a sample was dissolved in the CHCA matrix and tapped off the plate): MALDI TOF/TOF *m/z* $[M+Na]^+$ calcd for $C_{18}H_{31}N_3O_{11}$: 488.1856, found 488.1860.

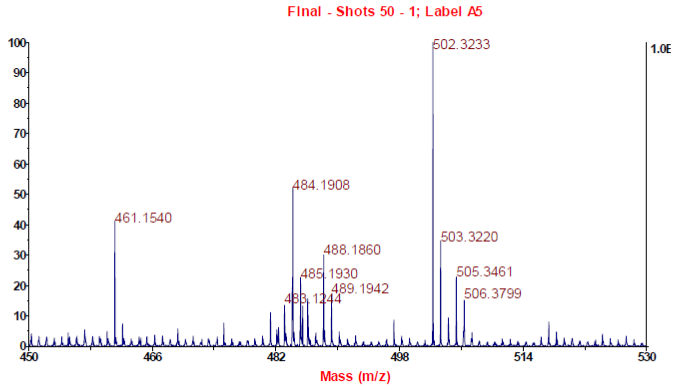

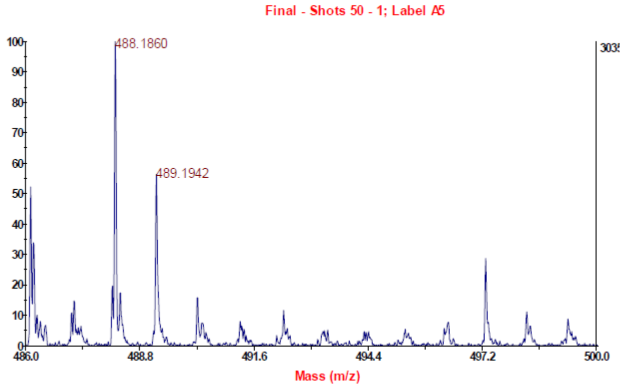

*N-(Adamantan-1-yl)-[(R)-3-(α-*D*-mannopyranosyloxy)-2-methylpro-panoyl]-*L*-alanyl-*D*-isoglutamine 5-amide* (**16**)

Starting from compound **14** (140 mg, 0.15 mmol), pure product **16** as colorless powder was obtained (72 mg, 83%): R_f=0.44 (CH_3CN/ H_2O 5:1); $[\alpha]_D^{25}$=+34.2 (c=0.8 in MeOH); ^{1}H NMR (600 MHz, CD_3OD) δ=4.74 (d, $J_{1,2}$=1.4 Hz, 1H, H-1), 4.32-4.27 (m, 2H, CH Ala, CH isoGln), 3.85 (dd, J=2.2, 11.7 Hz, 1H, H-6b), 3.77 (dd, $J_{1,2}$=1.8, $J_{2,3}$=3.6 Hz, 1H, H-2), 3.74-3.69 (m, 2H, H-6a, H-4), 3.68 (dd, $J_{2,3}$=3.32; $J_{3,4}$=9.3 Hz, 1H, H-3), 3.63-3.52 (m, 3H, CH_2 linker, H-5), 2.80-2.72 (m, 1H, CH linker), 2.20-2.16 (m, 2H, γ-CH_2 isoGln), 2.16-2.11 (m, 1H, β-CH isoGln), 2.06 (s, 3H, CH Ad), 2.04 (s, 6H, CH_2 Ad), 1.92-1.84 (m, 1H, β-CH isoGln), 1.73 (bs, 6H, CH_2 Ad), 1.40 (d, J=7.0 Hz, 3H, CH_3 Ala), 1.12 (d, J=7.0 Hz, 3H, CH_3 linker) ppm; ^{13}C NMR (150 MHz, CD_3OD) δ=177.70, 176.44, 175.41, 174.10 (C=O), 102.21 (C-1), 74.85, 72.66, 72.12, 68.70 (C-2, C-3, C-4, C-5), 71.20, 63.04 (C-6, CH_2 linker), 53.90 (CH isoGln), 52.91 (C Ad), 51.15 (CH Ala), 42.36 (CH_2 Ad), 41.97 (CH linker), 37.56 (CH_2 Ad), 34.11 (γ-CH_2 isoGln), 30.70 (CH Ad), 29.09 (β-CH_2 isoGln), 17.71 (CH_3 Ala), 14.45 (CH_3 linker)

ppm; HPLC: t_R=3.87 min; HRMS calcd for for $[C_{28}H_{46}N_4O_{10}+Na]^+$ 621.3112, found: 621.3065.

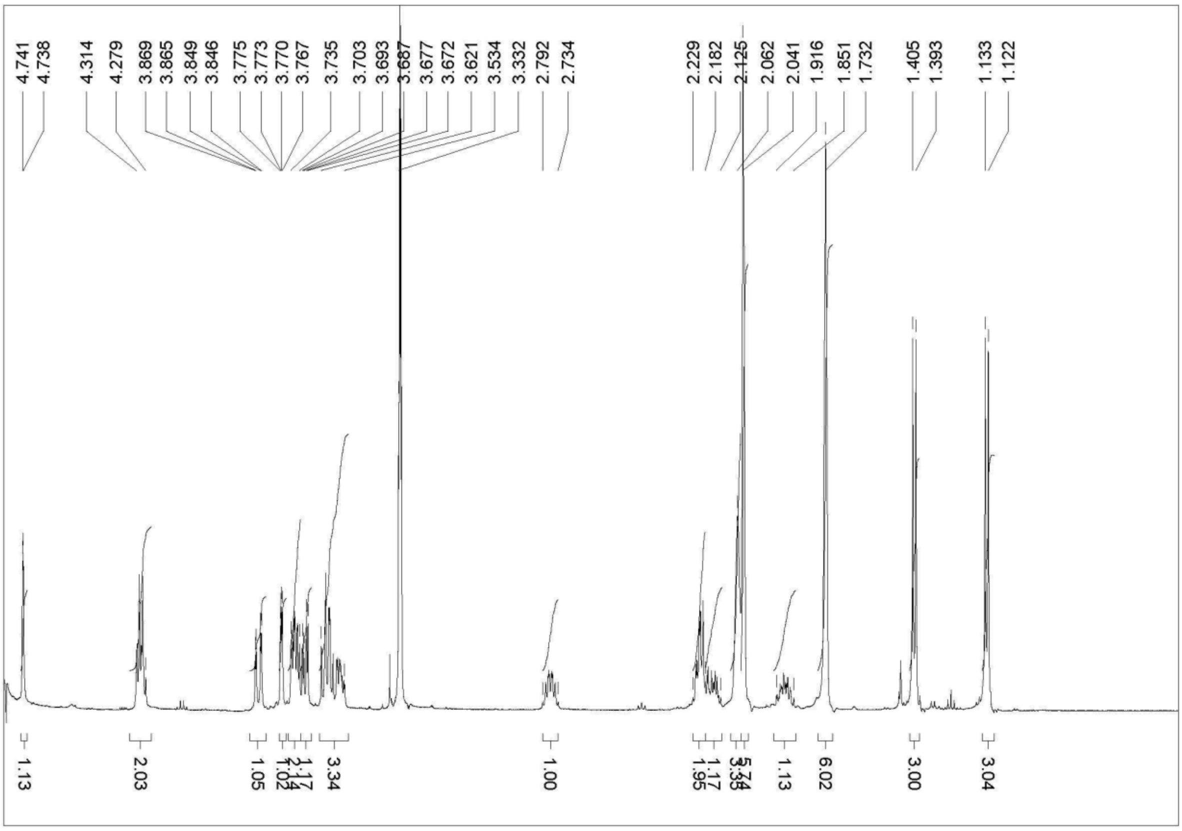

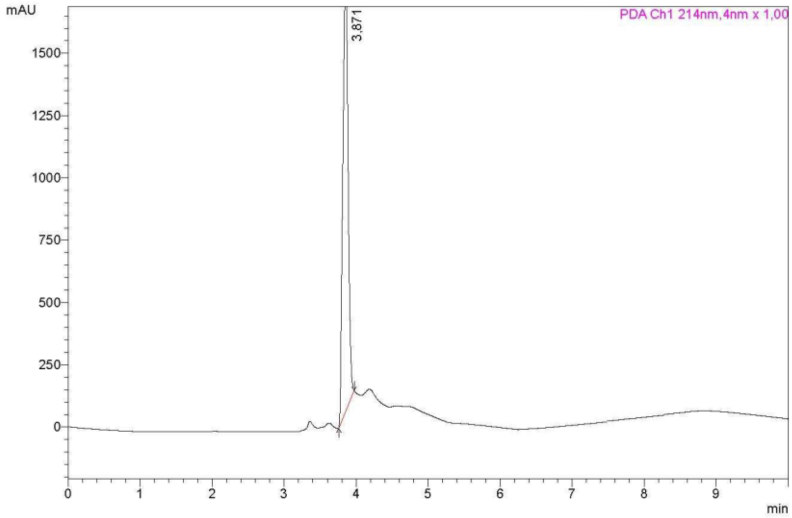

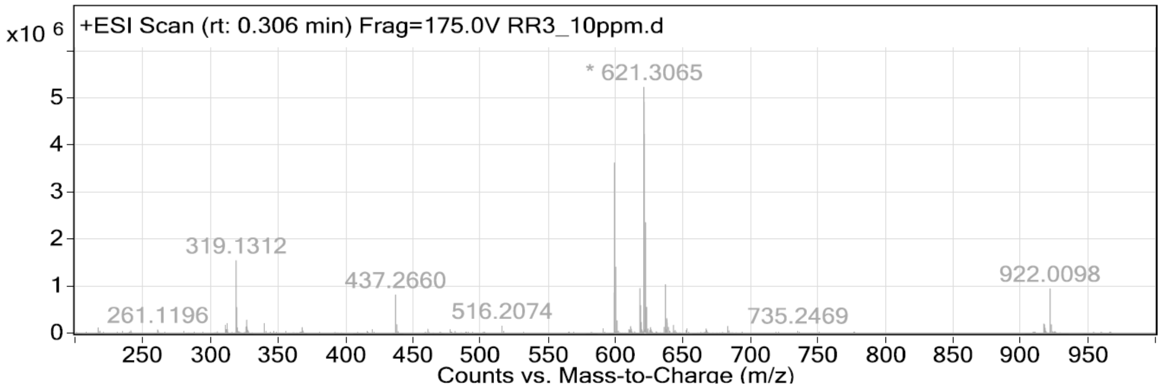

[2-(2,3,4,6-Tetra-O-acetyl-α-D-mannopyranosyloxy)ethanoyl]-L-alanyl-D-isoglutamine benzyl ester (**17**)

Starting from compound **1** (100 mg, 0.326 mmol) pure product **17** as yellow viscous oil was obtained (124 mg, 55%): R_f =0.73 ($CHCl_3$/MeOH 5:1); ^{1}H NMR (300 MHz, CD_3OD) δ=7.36–7.30 (m, 5H, CH_{arom} Bn), 5.41–5.36 (m, 2H, H-2, H-3), 5.26 (pt, J=9.7 Hz, 1H, H-4), 5.13 (s, 2H, CH_2 Bn), 4.93 (s, 1H, H-1), 4.39 – 4.32 (m, 2H, CH Ala, isoGln), 4.28 – 4.07 (m, 5H, H-5, H-6, OCH_2), 2.49 (pt, J=7.4 Hz, 2H, γ-CH_2 *iso*Gln), 2.30 – 2.19 (m, 1H, β-CH isoGln), 2.14 (s, 2H, CH_3 Ac), 2.05 (s, 2H, CH_3 Ac), 2.03 (s, 3H, CH_3 Ac), 1.97 (s, 3H, CH_3 Ac), 1.98–1.88 (m, 1H, β-CH isoGln), 1.39 (d, J=7.1 Hz, 3H, CH_3 Ala) ppm; ^{13}C NMR (75 MHz, CD_3OD) δ=176.22, 175.16, 174.34, 172.43, 171.61, 171.58, 171.55, 171.35 (C=O); 129.6, 129.31, 129.27 (CH_{arom} Bn), 99.26 (C1), 70.68, 70.47, 70.38, 67.17 (C2, C3, C4, C5), 67.54, 67.43, 63.45 (C6, CH_2 Bn, CH_2 linker), 53.78 (CH isoGln), 50.69 (CH Ala), 31.54 (γ-CH_2 isoGln), 27.87 (ß-CH_2 isoGln), 20.70, 20.67, 20.65, 20.63 (CH_3 Ac), 17.80 (CH_3 Ala) ppm; HRMS calcd for for $[C_{31}H_{41}N_3O_{15}+Na]^+$ 718.2436, found: 718.2439.

[2-(2,3,4,6-Tetra-O-acetyl-α-D-mannopyranosyloxy)ethanoyl]-D-(adamant-1-yl)glycyl-L-alanyl-D-isoglutamine benzyl ester (**18**)

Starting from compound **4** (41 mg, 0.069 mol) pure product **18** as yellow viscous oil was obtained (42 mg, 67%): R_f =0.30 ($CHCl_3$/MeOH 12:1); ^{1}H NMR (300 MHz, CD_3OD) δ=7.36–

7.29 (m, 5H, CH_{arom} Bn), 5.38–5.24 (m, 3H, H-2, H-3, H-4), 5.12 (s, 2H, CH_2 Bn), 4.93 (d, *J*=1.4 Hz, 1H, H-1), 4.37 (dd, *J*=9.9, *J*=4.5 Hz, 1H, CH isoGln), 4.33 – 4.01 (m, 5H, CH Ala, H-5, H-6, OCH_2), 2.46-2.41 (m, 2H, γ-CH_2 *iso*Gln), 2.27 – 2.17 (m, 1H, β-CH isoGln), 2.17 (s, 2H, CH_3 Ac), 2.05 (s, 2H, CH_3 Ac), 2.02 (s, 3H, CH_3 Ac), 2.00 (s, 3H, CH_3 Ac), 2.05–2.00 (m, 1H, β-CH isoGln), 1.37 (d, *J*=7.3 Hz, 3H, CH_3 Ala) ppm; ^{13}C NMR (75 MHz, CD_3OD) δ=176.29, 175.05, 174.28, 172.34, 172.19, 171.52, 171.46, 171.42, 171.03 (C=O), 129.59, 129.23, 129.21 (CH_{arom} Bn), 98.74 (C1), 70.69, 70.65, 70.24, 67.03 (C2, C3, C4, C5), 67.39, 66.98 (C6, CH_2 Bn), 53.81 (CH isoGln), 50.84 (CH Ala), 40.05, 37.80, 36.87 (CH_2 Ad), 31.54 (γ-CH_2 isoGln), 28.02 (ß-CH_2 isoGln), 29.82 (CH Ad), 20.69, 20.64, 20.65, 20.61 (CH_3 Ac), 17.847 (CH_3 Ala) ppm; HRMS calcd for for $[C_{43}H_{58}N_4O_{16}+Na]^+$ 909.3747, found: 909.3748.

N-(Adamantan-1-yl)-[2-(2,3,4,6-tetra-O-acetyl-α-D-mannopyrano-syloxy)ethanoyl]-L-alanyl-D-isoglutamine 5-amide (**19**)

Starting from compound **7** (158 mg, 0.341 mol) pure product **19** as yellow viscous oil was obtained (66 mg, 26%): R_f =0.71 ($CHCl_3$/MeOH 5:1); ^{1}H NMR (300 MHz, CD_3OD) δ=7.31 (s, 1H, CH), 5.40-5.30 (m, 2H, H-2, H-3), 5.26 (pt, *J*=9.7 Hz, 1H, H-4), 4.93 (s, 1H, H-1), 4.35 (q, *J*=7.1 Hz, 1H, CH Ala), 4.39–4.08 (m, 6H, *iso*Gln, H-5, H-6, OCH_2), 3.75–3.63 (m, 1H, OCH), 2.45–2.36 (m, 1H, γ-CH isoGln), 2.22–2.14 (m, 3H, γ-CH isoGln, β-CH_2 isoGln), 2.14 (s, 3H, CH_3 Ac), 2.06–2.02 (m, 15H; Ad), 1.97 (s, 2H, CH_3 Ac), 1.71 (s, 6H, CH_3 Ac), 1.43 (d, *J*=7.1 Hz, 3H, CH_3 Ala) ppm; ^{13}C NMR (75 MHz, CD_3OD) δ=176.49, 175.10, 174.22, 172.42, 171.61, 171.58, 171.54, 171.48 (C=O), 99.28 (C1), 70.67, 70.49, 70.39, 67.18 (C2, C3, C4, C5), 67.52, 63.45(C6, CH_2 linker), 54.01 (CH isoGln), 50.90 (CH Ala), 53.01 (C Ad), 42.37, 37.57 (CH_2 Ad), 34.17 (γ-CH_2 isoGln), 28.93 (ß-CH_2 isoGln), 30.97 (CH Ad), 20.70, 20.68, 20.66, 20.63 (CH_3 Ac), 17.74 (CH_3 Ala) ppm; HRMS calcd for for $[C_{34}H_{50}N_4O_1+H]^+$ 739.3403, found: 739.3398.

[2-α-D-Mannopyranosyloxy)ethanoyl]-L-alanyl-D-isoglutamine (**20**)

Compound **17** (40 mg, 0.067 mol) was first subjected to hydrogenolysis and after without purification it was deacetylated giving pure product **20** was obtained in the form of viscous oil (26 mg, 89%): R_f =0.31 ($CHCl_3$/MeOH 1:1); ^{1}H NMR (300 MHz, CD_3OD) δ=4.85 (d, J=1.6 Hz, 1H, H-1), 4.42 (q, J=7.0 Hz, 1H, CH Ala), 4.27–4.24 (dd, J=9.4, J=4.3 Hz, 1H, CH isoGln), 4.23-4.06 (m, 2H, H-3, H-4), 3.97 – 3.96 (dd, J=3.4, J =1.7 Hz, 1H, H-2), 3.86–3.54 (m, 5H, H-5, H-6, OCH_2), 2.30 – 2.26 (m, 2H, γ-CH_2 isoGln), 2.17–2.08 (m, 1H, β-CH isoGln), 1.99–1.90 (m, 1H, β-CH isoGln), 1.41 (d, J=7.1 Hz, 3H, CH_3 Ala) ppm; ^{13}C NMR (75 MHz, CD_3OD) δ=181.36, 177.02, 174.88, 171.78 (C=O), 101.76 (C1), 75.35, 72.38, 71.73, 68.66 (C2, C3, C4, C5), 66.92, 62.88 (C6, CH_2 linker), 55.18 (CH isoGln), 50.39 (CH Ala), 35.13 (γ-CH_2 isoGln), 29.31 (ß-CH_2 isoGln), 18.22 (CH_3 Ala) ppm; HPLC: t_R=2.39 min; HRMS calcd for for $[C_{16}H_{27}N_3O_{11}+Na]^+$ 460.1544, found: 460.1541.

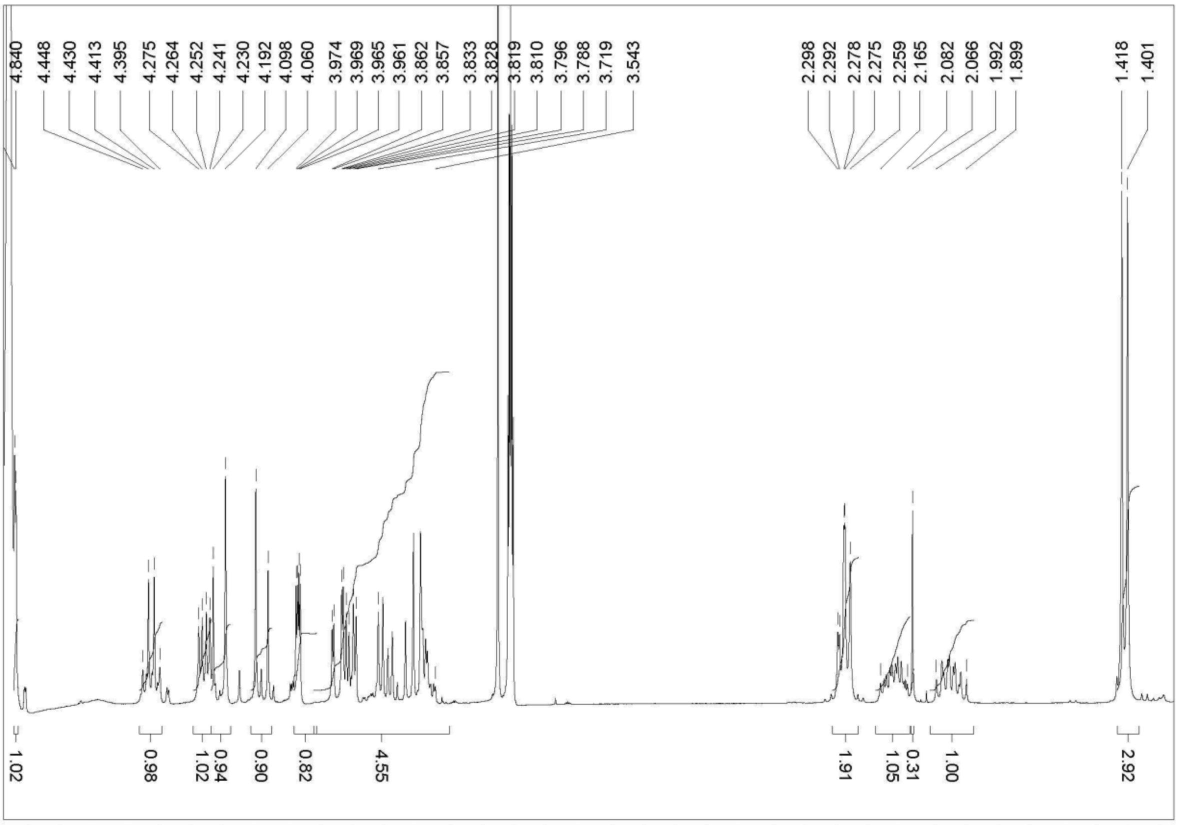

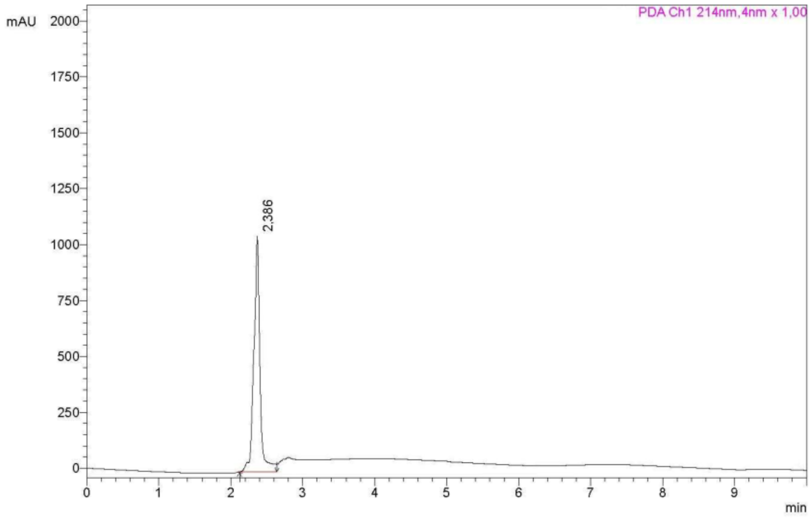

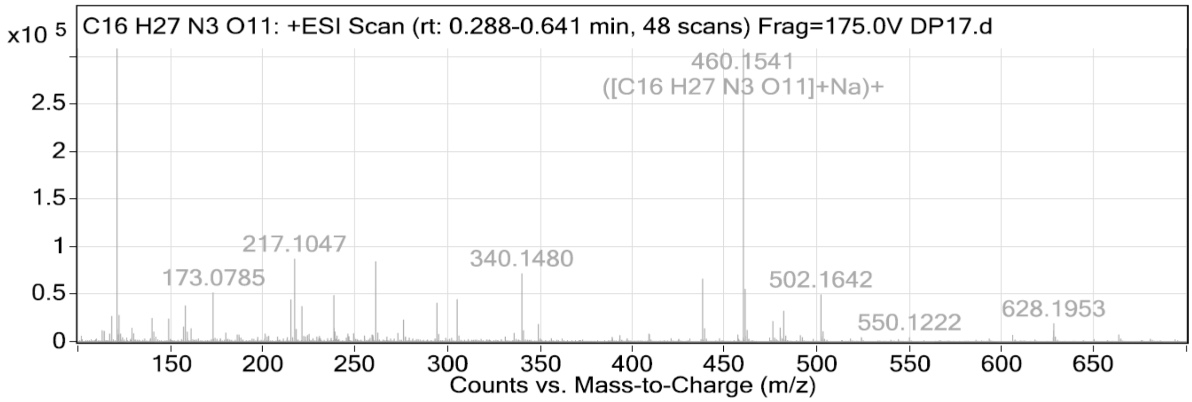

[2-α-D-Mannopyranosyloxy)ethanoyl]-D-(adamant-1-yl)glycyl-L-alanyl-D-isoglutamine (**21**)

Compound **18** (34 mg, 0,038 mmol) was first subjected to hydrogenolysis and after without purification it was deacetylated giving pure product **21** in the form of viscous oil (14 mg, 59 %): R_f =0.42 ($CHCl_3$/MeOH 1:1); ^{1}H NMR (300 MHz, CD_3OD) δ=4.84 (d, *J*=1.6Hz, 1H, H-1), 4.33-4.23 (m, 2H, CH Ala, CH isoGln), 4.16–4.08 (m, 2H, H-3, H-4), 3.94 (dd, *J*=3.3, *J*=1.8 Hz, 1H, H-2), 3.84 (dd, *J*=2.3, *J*=11.8 Hz, 1H, H-6a), 3.77–3.52 (m, 4H, H-5, H-6b, OCH_2), 2.27 (pt, *J* =7.6, *J*=7.1 Hz, 2H, γ-CH_2 isoGln), 2.19–2.11 (m, 1H, β-CH isoGln), 2.00–1.96 (m, 3H, Ad), 1.96–1.90 (m, 1H, β-CH isoGln), 1.73–1.66 (m, 9H, Ad), 1.60–1.57 (m, 3H, Ad), 1.38 (d, *J*=7.2 Hz, 3H, CH_3 Ala) ppm; ^{13}C NMR (75 MHz, CD_3OD) δ=181.03, 177.07, 175.15, 171.80, 171.52 (C=O), 101.68 (C1), 75.60, 72.56, 71.71, 68.42 (C2, C3, C4, C5), 66.95, 62.86 (C6, CH_2 linker), 62.45 (CH AdGly), 54.89 (CH isoGln), 50.69 (CH Ala),

49.90 (C Ad), 40.02, 37.86, 37.68 (CH_2 Ad), 34.75(γ-CH_2 isoGln), 29.20 (ß-CH_2 isoGln), 29.83 (CH Ad), 17.63 (CH_3 Ala) ppm; HPLC: t_R=2.31 min; HRMS calcd for for $[C_{28}H_{244}N_4O_{12}+Na]^+$ 651.2857, found: 651.2831.

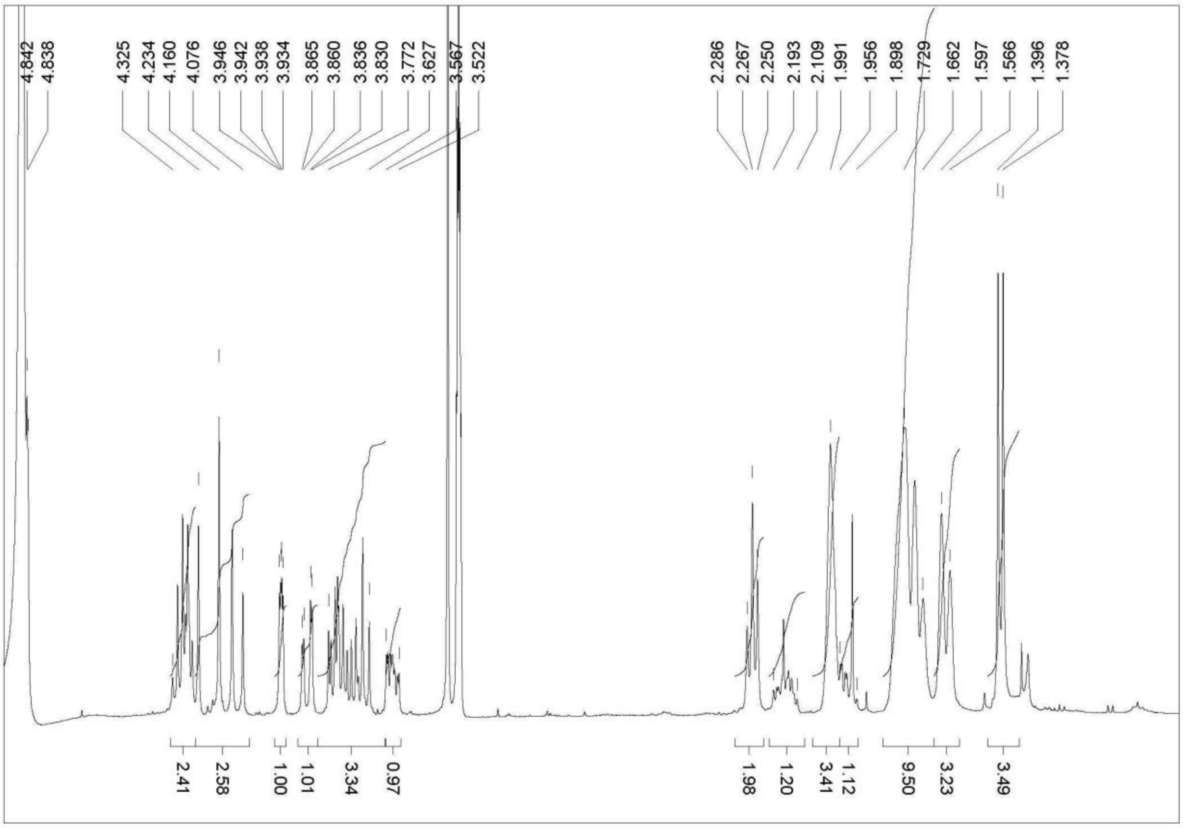

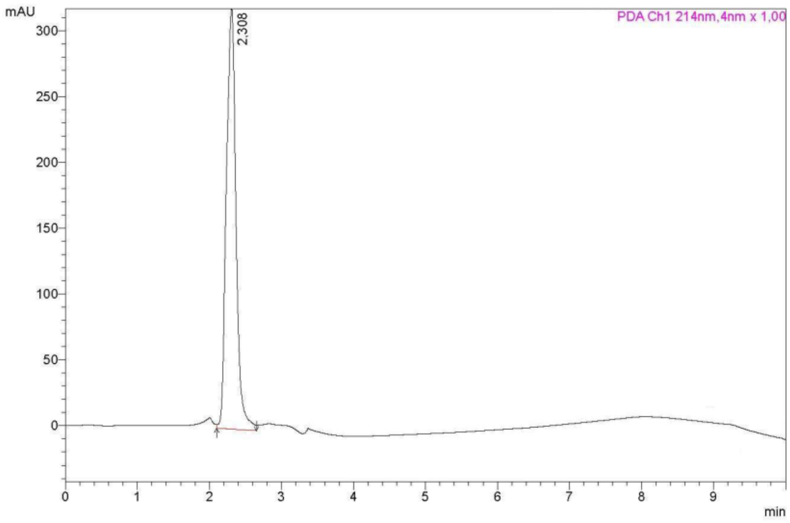

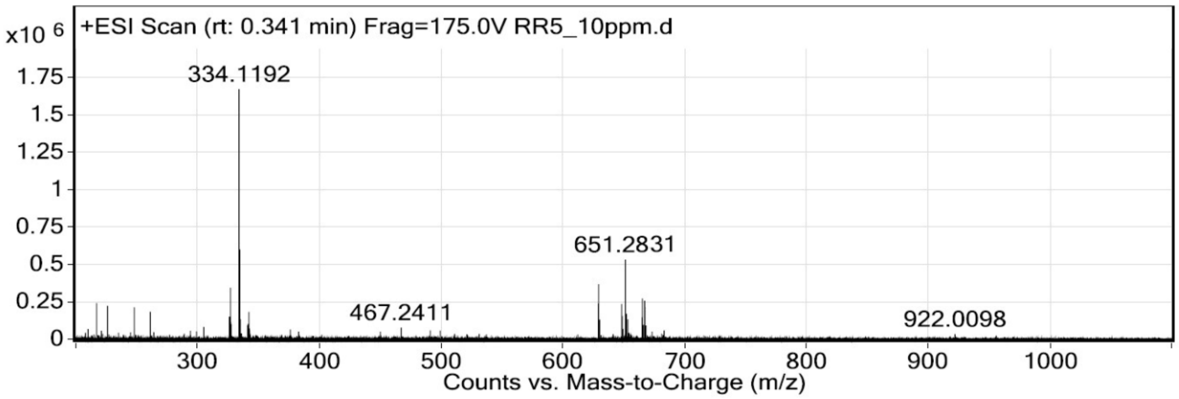

N-(Adamantan-1-yl)-[2-α-D-mannopyranosyloxy)ethanoyl]-L-alanyl-D-isoglutamine 5-amide **22**

Starting from compound **19** (55 mg, 0.074 mmol), pure product **22** was obtained in the form of viscous oil (33 mg, 77 %): R_f =0.62 ($CHCl_3$/MeOH 1:1); ^{1}H NMR (300 MHz, CD_3OD) δ=4.84 (d, *J*=6.3 Hz, 1H, H-1), 4.39 (q, *J*=7.1 Hz, 1H, CH Ala), 4.30 (dd, *J*=9.4, *J*=4.4 Hz, 1H, CH isoGln), 4.25–4.08 (m, 2H, H-3, H-4), 3.96–3.95 (dd, *J*=3.4, *J*=1.7 Hz, 1H, H-2), 3.86–3.52 (m, 5H, H-5, H-6, OCH_2), 2.22–2.18 (pt, *J* =7.1, *J*=7.5 Hz, 2H, γ-CH_2 isoGln), 2.16–2.08 (m, 1H, β-CH isoGln), 2.05–2.02 (m, 9H, Ad), 1.93–1.83 (m, 1H, β-CH isoGln), 1.71 (s, 6H, Ad), 1.42 (d, *J*=7.1 Hz, 3H, CH_3 Ala) ppm; ^{13}C NMR (75 MHz, CD_3OD) δ=176.45, 174.92, 174.09, 171.97 (C=O), 101.81 (C1), 75.44, 72.45, 71.75, 68.62 (C2, C3, C4, C5), 66.91, 62.94 (C6, CH_2 linker), 54.02 (CH isoGln), 50.57 (CH Ala), 52.90 (C Ad), 42.34, 37.56 (CH_2 Ad), 34.08 (γ-CH_2 isoGln), 29.00 (ß-CH_2 isoGln), 30.95 (CH Ad), 18.06 (CH_3 Ala) ppm; HPLC: t_R=2.82 min; HRMS calcd for for $[C_{26}H_{42}N_4O_{10}+Na]^+$ 593.2799, found: 593.2800.

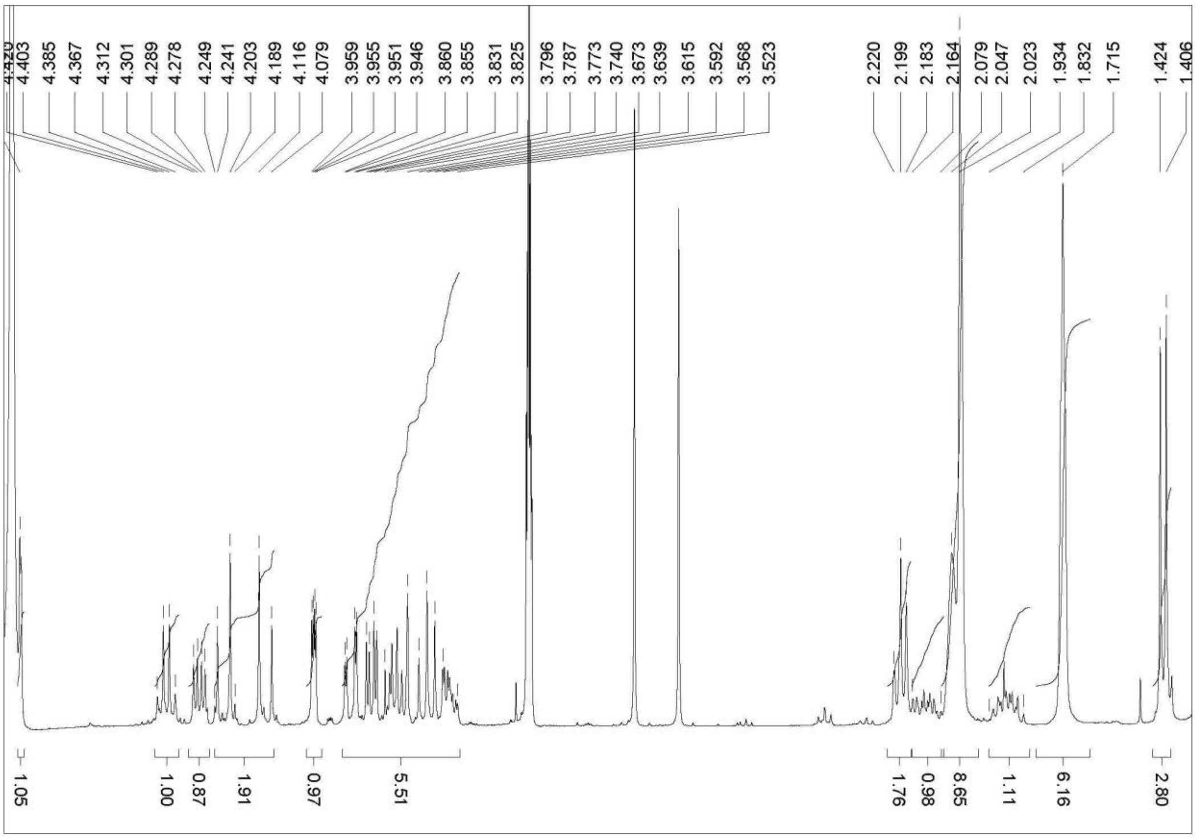
4.403
4.385
4.367
4.312
4.301
4.289
4.278
4.249
4.241
4.203
4.189
4.116
4.079
3.959
3.955
3.951
3.946
3.860
3.855
3.831
3.825
3.796
3.787
3.773
3.740
3.673
3.639
3.615
3.592
3.568
3.523
2.220
2.199
2.183
2.164
2.079
2.047
2.023
1.934
1.832
1.715
1.424
1.406
1.05
1.00
0.87
1.91
0.97
5.51
1.76
0.98
8.65
1.11
6.16
2.80

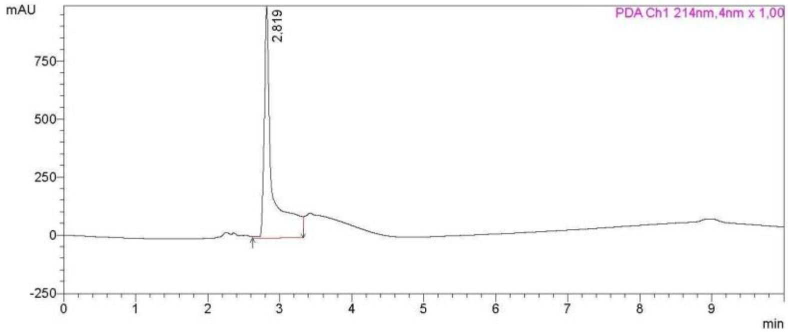
mAU
PDA Ch1 214nm,4nm x 1,00
2.819
750
500
250
0
-250
min

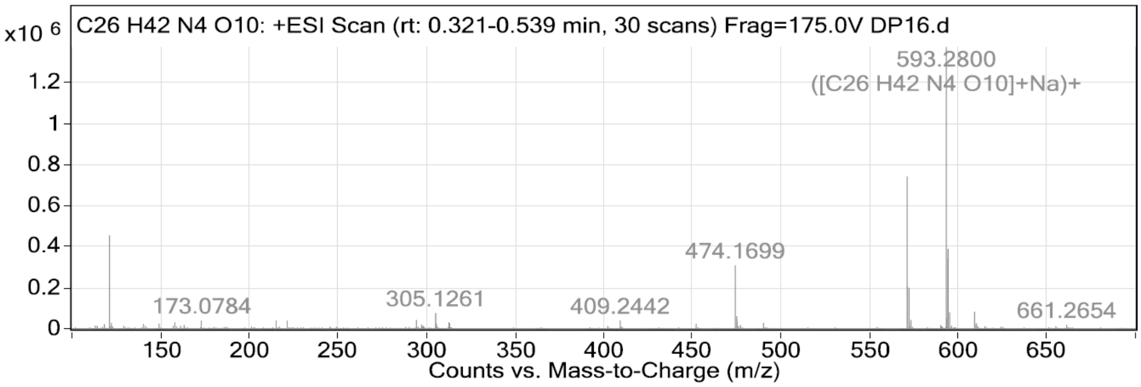
C26 H42 N4 O10: +ESI Scan (rt: 0.321-0.539 min, 30 scans) Frag=175.0V DP16.d
x10 6
593.2800
([C26 H42 N4 O10]+Na)+
474.1699
173.0784
305.1261
409.2442
661.2654
Counts vs. Mass-to-Charge (m/z)

Experiment in vivo

Healthy, nulliparous and non-pregnant BALB/c female mice, 8–12 weeks of age at the initiation of the experiment were used. Mice were obtained from the Ruđer Bošković Institute's breeding colony. During experimental period groups of five animals was kept per cage. Bottom of the cage was covered with sawdust (Scobis Uno®, Muchedola srl Italy). Standard food for laboratory mice (4RF 21 GLP® Mucedola srl, Italy) was used. Access to food and water was ad libitum. Animals were kept in conventional circumstances: light/dark rhythms 12/12 h, temperature 22 °C, and humidity 55%. All experiments were performed according to ILAR Guide for the Care and Use of Laboratory Animals, EU Directive 2010/63/EU and Croatian animal protection law (NN 102/17). Experimental groups of five mice were immunized and boosted two times subcutaneously (s.c.) into the tail base at 21-days intervals. Mice were anesthetised by Isoflurane prior to blood collection on the 7th day after the second booster. Sera were collected, decomplemented at 56 °C for 30 min and stored at −20 °C until tested. The dose of OVA (antigen) was 10 µg per mouse. The dose of PGM and tested compounds was 200 µg per mouse. OVA and tested substances were dissolved in water and the injection volume in all experimental groups was 0.1 ml per mouse.

Measurements of antibody

Enzyme immunoassays (ELISA) were performed on flat-bottomed high binding microtitre plates (*Costar*, USA) using mouse anti-OVA IgG, IgG1 and IgG2a antibody assay kits (*Chondrex*, USA), according to the manufacturer's instructions. The ratio of anti-OVA IgG1 and anti-OVA IgG2a (IgG1/IgG2a) was used as indication of the Th1/Th2-bias of induced immune response.

Statistics

Statistical analyses were performed using GraphPad Prism Software. The significant difference between experimental groups was evaluated by Kruskal-Wallis ANOVA, followed by Dunn's multiple comparison test. A probability values less than 0.05 ($p < 0.05$) were considered significant.
